# A new mechanism for cannabidiol in regulating the one‐carbon cycle and methionine levels in *Dictyostelium* and in mammalian epilepsy models

**DOI:** 10.1111/bph.14892

**Published:** 2020-01-03

**Authors:** Christopher J. Perry, Paul Finch, Annette Müller‐Taubenberger, Kit‐Yi Leung, Eleanor C. Warren, Joseph Damstra‐Oddy, Devdutt Sharma, Pabitra H. Patra, Sarah Glyn, Joanna Boberska, Balint Stewart, Amy Baldwin, Fabiana Piscitelli, Robert J. Harvey, Adrian Harwood, Christopher Thompson, Sandrine P. Claus, Nicholas D.E. Greene, Alister J. McNeish, Claire M. Williams, Benjamin J. Whalley, Robin S.B. Williams

**Affiliations:** ^1^ Centre for Biomedical Sciences, Department of Biological Sciences Royal Holloway University of London Egham UK; ^2^ Department of Cell Biology Biomedical Center, LMU Munich Planegg Germany; ^3^ Development Biology and Cancer Program UCL Great Ormond Street Institute of Child Health London UK; ^4^ The School of Chemistry, Food Biosciences and Pharmacy University of Reading Reading UK; ^5^ Faculty of Life Sciences Manchester University Manchester UK; ^6^ Neuroscience and Mental Health Research Institute Cardiff University Cardiff UK; ^7^ Institute of Biomolecular Chemistry Consiglio Nazionale delle Ricerche Rome Italy; ^8^ School of Health and Sport Sciences University of the Sunshine Coast Sippy Downs QLD Australia; ^9^ Sunshine Coast Health Institute University of the Sunshine Coast Birtinya QLD Australia

## Abstract

**Background and Purpose:**

Epidiolex™, a form of highly purified cannabidiol (CBD) derived from *Cannabis* plants, has demonstrated seizure control activity in patients with Dravet syndrome, without a fully elucidated mechanism of action. We have employed an unbiased approach to investigate this mechanism at a cellular level.

**Experimental Approach:**

We use a tractable biomedical model organism, *Dictyostelium*, to identify a protein controlling the effect of CBD and characterize this mechanism. We then translate these results to a Dravet syndrome mouse model and an acute in vitro seizure model.

**Key Results:**

CBD activity is partially dependent upon the mitochondrial glycine cleavage system component, GcvH1 in *Dictyostelium*, orthologous to the human glycine cleavage system component H protein, which is functionally linked to folate one‐carbon metabolism (FOCM). Analysis of FOCM components identified a mechanism for CBD in directly inhibiting methionine synthesis. Analysis of brain tissue from a Dravet syndrome mouse model also showed drastically altered levels of one‐carbon components including methionine, and an in vitro rat seizure model showed an elevated level of methionine that is attenuated following CBD treatment.

**Conclusions and Implications:**

Our results suggest a novel mechanism for CBD in the regulating methionine levels and identify altered one‐carbon metabolism in Dravet syndrome and seizure activity.

**Abbreviations**
5mTHF: 5′methyl THF
bsR: blasticidin S deaminase
CBD: cannabidiol
FOCM: folate one‐carbon metabolism
GCS: glycine cleavage system
GCSH: human glycine cleavage system component H
GCVH1: *Dictyostelium* glycine cleavage system component H1
THF: tetrahydrofolate

**What is already known**

- CBD, a key constituent of Epidiolex™, is effective in the treatment of specific drug‐resistant epilepsies.
- A full understanding of mechanisms for CBD‐dependent s eizure control remains to be established.

**What does this study add**

- We identified a molecular mechanisms of CBD in regulating the one‐carbon cycle components including methionine.
- We show one‐carbon cycle components are deregulated in a Dravet model and during seizure‐like activity.

**What is the clinical significance**

- Our data will increase clinical focus on one‐carbon metabolism in epilepsy and other CBD‐treatable disorders.
- Clinical studies relating to Dravet syndrome, in particular, will benefit from monitoring one‐carbon signalling.

## 1 INTRODUCTION

The use of *Cannabis* extracts as a medicinal treatment has been recorded for nearly 2,000 years (BRAND & ZHAO, 2017), covering a range of disorders including pain management, multiple sclerosis, and epilepsy (Devinsky et al., 2014). Of the ~100 cannabinoids found within, or derived from, *Cannabis* species, cannabidiol (CBD) is among the most abundant of the non‐psychoactive cannabinoids and has received considerable interest as a therapeutic treatment (Jones et al., 2010; Jones et al., 2012). CBD has been shown to provide antiepileptiform and anti‐seizure properties within numerous in vitro and in vivo mammalian epilepsy models (Jones et al., 2012; Klein et al., 2017). CBD is also used as a lead cannabinoid‐based treatment for severe epilepsies such as Dravet syndrome (Devinsky et al., 2017) and Lennox–Gastaut syndrome (Devinsky et al., 2018; Thiele et al., 2018). A range of targets for CBD in seizure control have also been identified, including glycine receptors (Xiong et al., 2012), GPR55 (Kaplan, Stella, Catterall, & Westenbroek, 2017), NMDA receptors (Rodriguez‐munoz, Onetti, Cortes‐Montero, Garzon, & Sanchez‐Blazquez, 2018), transient receptor potential of vanilloid type‐1 channels (Vilela et al., 2017) and voltage‐dependent anion selective channels (Rimmerman et al., 2013).

The cell and molecular basis of epilepsy and seizures is well‐accepted to be related to electrical signalling and ion channel activity; however, a range of models have suggested that deregulation of several key amino acids is also involved in, or associated with, seizures and epilepsy (Bejarano & Rodriguez‐navarro, 2015; Gupta et al., 2004). For example, numerous studies have proposed altered mitochondrial function in epilepsy pathology (Doccini et al., 2015; Kumar et al., 2016; Panneman, Smeitink, & Rodenburg, 2018; Pearson‐smith, Liang, Rowley, Day, & Patel, 2017). Mitochondria contain the glycine cleavage system (GCS) that is responsible for regulation of glycine levels (Kikuchi & Hiraga, 1982) subsequently controlling numerous functions including protein synthesis and neurotransmission (Kolker, 2018). The GCS also plays a role in providing glycine‐derived one‐carbon units into the folate one‐carbon metabolism (FOCM), leading through folate‐containing intermediates, to production of methionine from homocysteine (Ducker & Rabinowitz, 2017). Isotope labelling has directly demonstrated 1C donation from glycine to FOCM in humans and mice in vivo (Lamers et al., 2009; Leung et al., 2017). Methionine fulfils a fundamentally important role in protein synthesis and as a precursor to S‐adenosyl methionine (SAM) that is responsible for the transfer of a methyl group to a number of acceptor molecules including neurotransmitters and DNA methyltransferases (Moore, Le, & Fan, 2013). SAM is further hydrolysed to form adenosine and homocysteine which can again be converted into methionine or instead into cysteine. These various metabolites and amino acids have been described as “primordial” metabolites (Boison, 2016), suggested to provide the original components for the beginning of life, and are thus highly conserved across many species. In addition, many of these components are regulated by mitochondrial function and relate to energy production, where both have been proposed as mechanistic targets for cannabinoids (Bénard et al., 2012).

Understanding the molecular mechanisms of newly developed medicines provides an important aspect of validation, where simple biomedical model systems such as the social amoeba *Dictyostelium discoideum* have proved effective (Cunliffe et al., 2015; Warren, Walker, & Williams, 2018). *D.* *discoideum* is a eukaryote more evolutionarily related to animals than to plants and bacteria that possesses a range of highly conserved signalling pathways and proteins that have been linked to human diseases (Müller‐taubenberger, Kortholt, & Eichinger, 2013). It exists in both a single cell stage, where cells divide by binary division, and a multicellular stage induced by starvation leading to the formation of small (~1 mm tall) fruiting bodies. Using *D.* *discoideum*, insertional mutant libraries have been employed to identify genes controlling the effect of a natural product or medicinal treatment (Cocorocchio et al., 2018; Waheed et al., 2014), including treatments for epilepsy (Chang et al., 2012; Warren et al., 2018) and bipolar disorder (Kelly, Sharma, Wilkinson, & Williams, 2018; Williams, Cheng, Mudge, & Harwood, 2002). In many of these studies, discoveries based in *D.* *discoideum* have been subsequently validated in mammalian models (Chang et al., 2013; Chang et al., 2015; Chang, Walker, & Williams, 2014; Waheed et al., 2014). Thus, employing *D.* *discoideum* to investigate the cellular mechanisms of CBD may provide a suitable, innovative approach to identify therapeutic mechanisms of this important cannabinoid.

In the initial phase of this study, we used *D.* *discoideum* to better understand the molecular mechanism of CBD. We show that CBD affects *D.* *discoideum* cell proliferation and by performing an unbiased genome wide screen, we identify a role for the mitochondrial GCS component, GCVH1, homologous to the human protein GSCH in this effect. Analysis of glycine, one‐carbon cycle intermediates (methionine and cysteine), and folates confirmed a role for GCVH1 in regulating these components through attenuating activity of the GCS and suggested a novel molecular mechanism of CBD through inhibition of methionine synthase activity. We then examined the regulation of one‐carbon‐related amino acids in the brain of a mouse model of Dravet syndrome (*Scn1A* ^+/−^), a CBD responsive condition in humans (Thiele et al., 2018), and identified altered levels of glycine, methionine, and cysteine, implicating dysregulation of one‐carbon metabolism in this disease. Moreover, methionine levels were altered in this model following chronic CBD treatment. We finally investigated this effect using an in vitro mammalian model of seizure activity, where methionine levels were shown to rapidly increase during seizure activity and this increase was corrected by CBD treatment.

## 2 METHODS

### 2.1 *D*. *discoideum* growth assay

*D.* *discoideum* cell lines grown in HL5 medium on a 10‐cm dish were plated onto a 24‐well plate at 5 × 10^3^ cells per well in 495 μl of media (HL5, Formedia, UK). For each well, 5 μl of CBD from 100‐mM stock in DMSO was added to achieve final concentrations ranging from 0‐ to 20‐μM CBD (DMSO made up 1% of final volume). Cells were maintained at 22°C, counted after 72 hr (lag phase), and then every 24 hr. These data are presented as normalized (fold change from control [untreated, Day 7]) to correct for variation between independent experiments. Each concentration was analysed using at least six independent experiments, where rate of exponential cell growth (Days 4 to 6) at each concentration was used to create secondary plots (concentration–response curves), providing IC_50_ values for each cell type (wild type, mutants, and rescue lines), determined using non‐linear regression analysis.

### 2.2 *D*. *discoideum* development assay

Wild‐type *D.* *discoideum* cells in the exponential phase of growth were washed in KK2 buffer (16.2‐mM KH_2_PO_4_, 4‐mM K_2_HPO_4_) and 1 × 10^7^ cells placed onto a nitrocellulose filter (Millipore, Cork). Absorbent pads (Millipore, Cork) were placed in 2‐ml culture dishes and soaked with 0.5‐ml KK2 buffer containing CBD from 100‐mM stock in DMSO to achieve a final concentration of 20 μM (DMSO made up 1% of final volume). Nitrocellulose filters and cells were placed upon absorbent pads and maintained in a humid environment at 22°C for 24 hr. Fruiting body morphology was recorded using a dissection microscope and camera.

### 2.3 Restriction enzyme‐mediated integration screen

A mutant library containing approximately 5,000 insertional mutants was created by restriction enzyme‐mediated integration. Library cells taken from the exponential growth phase were screened for resistance to 9.47‐μM CBD (80% inhibitory concentration) taken from 100‐mM stock in DMSO. Cells were maintained at 22°C and screened over a 3‐week period, with the media replaced every 2 days. Resistant mutants were isolated, and isogenic strains created. Location of the blasticidin S deaminase (bsR) insertion was identified using whole‐genome next‐generation sequencing (NGS).

### 2.4 NGS analysis

Five hundred‐nanogram DNA was fragmented on the Covaris S2 to a target size of 300 bp (intensity: 4, duty cycle: 10%, cycles‐per‐burst: 200, time: 80 s). A library was then prepared using the NEB DNA Ultra II kit (New England Biolabs, E7645), with bead selection for 300‐ to 400‐bp fragments and five cycles of PCR. Sequencing was performed by UCL Genomics on an Illumina NextSeq 500, using a 150‐bp paired‐end protocol. NGS assemblies were performed using Sequencher software (https://scicrunch.org/resources/Any/search?q=SCR_001528&l=SCR_001528), using Velvet (Zerbino & Birney, 2008) for de novo assembly, or GSNAP (https://scicrunch.org/resources/Any/search?q=SCR_005483&l=SCR_005483) for bsR‐cassette‐guided assembly, to reveal the sites of bsR‐cassette insertions in the *D.* *discoideum* genome using BLAST searches (https://scicrunch.org/resources/Any/search?q=SCR_001653&l=SCR_001653).

### 2.5 Creation of *gcvH1* ^*−*^ cell line and overexpression cell lines

In order to recapitulate the resistance seen in the insertional *gcvH1* ^−^ mutant, a *gcvH1* ^−^ cell line was created via homologous recombination. Primers to both the 5′ and 3′ region of *gcvH1* were used to amplify PCR products for both regions (Figure S1). A 281‐bp fragment was amplified from the 5′ region, while a 343‐bp fragment was amplified of the 3′ region (Figure S1). Both fragments were ligated into the pLPBLP plasmid flanking the bsR gene with their reading frames in opposite orientation to bsR. A knockout cassette consisting of bsR and the flanking *gcvH1* fragments was created by a *Spe*I/*Kpn*I restriction digest. Wild‐type cells were transformed with the digested plasmid by electroporation. Transformants were selected by growing in liquid medium containing 10 μg·ml^−1^ blasticidin and screened for homologous recombinants. Primers were designed that would produce diagnostic knockout fragments unique to the homologously integrated transformants, as well as the necessary controls (Figure S1).

To create a gcvH1 overexpression plasmid (Figure S1), the gcvH1 cDNA was cloned into an extra‐chromosomal vector (Fischer et al., 2004), enabling expression of the 42‐kDa fluorescently labelled protein (Figure S2). The PCR product was digested with *Eco*RI and *Bam*HI and then ligated into the extra‐chromosomal plasmid pDXA‐389‐2 and sequenced to confirm that no mutations were introduced. The resulting plasmid was transformed into *gcvH1* ^*−*^ cells and selected using geneticin (10 mg·ml^−1^) to create an overexpression cell line. A cDNA encoding the *Homo sapiens* orthologue human glycine cleavage system component H (https://www.guidetopharmacology.org/GRAC/ObjectDisplayForward?objectId=3098) was codon optimized for *D.* *discoideum* and amplified using primers 11 + 12. This was also inserted into pDXA‐389‐2 and a further overexpression cell line was created that overexpresses the human GCSH cDNA. In both cases, the recombinant proteins were tagged with RFP at the C‐terminus.

### 2.6 Analysis of *D*. *discoideum gcvH1* and *H*. *sapiens* *GCSH* expression by RT‐PCR

Total RNA was extracted from cell lines using an RNeasy mini‐kit (Qiagen). cDNA was synthesized from total RNA treated with DNase (Life Technologies) using a first‐strand cDNA synthesis kit (Thermo Scientific). Gene expression was confirmed via RT‐PCR using the primers 13 + 14 for the *D.* *discoideum* gene and primers 15 + 16 for the *H*. *sapiens* gene. A control was used based upon the constitutive expression of the *Ig7* housekeeping gene using primers 17 + 18.

### 2.7 Western blot analysis to confirm the presence of RFP fusion proteins in the overexpression cell lines

Cell lysates from cell lines were separated by gel electrophoresis, transferred to nitrocellulose membranes (Merck Millipore, IPFL00010), and analysed by Western blotting. A mouse anti‐RFP primary antibody (Chromotek, 6G6 anti‐RFP, 1:1,000) and a goat anti‐mouse secondary antibody (Li‐Cor goat anti‐mouse IRDye®, 1:1,000) were used to confirm the presence of RFP. A conjugated anti‐streptavidin antibody (Invitrogen, Streptavidin Alexa Fluor 680 conjugate) was used as a loading control. Blots were analysed using Odyssey software.

### 2.8 Fluorescence and live‐cell microscopy

The immuno‐related procedures used comply with the recommendations made by the *British Journal of Pharmacology* (Alexander et al., 2018). For immunolabelling, *gcvH1* ^*−*^ cells, expressing either *D.* *discoideum* gcvH1‐RFP or *H*. *sapiens* GCSH‐RFP, were plated on round 12‐mm glass coverslips and after 20 min were fixed with 15% picric acid/2% paraformaldehyde in 10‐mM PIPES, pH 6.0, for 20 min and postfixed with 70% ethanol for 10 min. Cells were then washed three times in PBS, once with 10‐mM PIPES and twice with PBS/1% glycine, and incubated in blocking buffer (PBS plus 2% BSA) for 1 hr at room temperature (RT). After blocking, the cells were washed three times with PBS and incubated with primary antibodies for 2 hr at RT, followed by the incubation with secondary antibodies and DAPI, to stain DNA, for 1 hr at RT. After immunostaining, samples were washed three times in PBS and embedded using Dako mounting medium (Agilent Technologies). For visualization of filamentous actin, cells were stained with Atto 488‐phalloidin (Sigma Aldrich). In order to visualize mitochondrial porin, cells were stained with mouse monoclonal anti‐porin antibodies (70‐100‐1; Troll et al., 1993), and Alexa 488‐conjugated goat anti‐mouse IgG. DNA was visualized by staining with DAPI. For live‐cell microscopy, cells were seeded in μ‐dishes (Ibidi, Germany) or open chambers as described previously (Fischer, Haase, Simmeth, Gerisch, & Muller‐Taubenberger, 2004).

Confocal microscopy was performed at the Bioimaging core facility of the Biomedical Center (LMU Munich) using an inverted Leica TCS SP8 equipped with lasers for 405‐, 488‐, 552‐, and 638‐nm excitation. Images were acquired with an HC PL APO 63×/1.40 oil PH3 objective. Recording was sequentially to avoid bleed through. Atto‐488 and Alexa‐488 and RFP were recorded with the hybrid photo detectors and DAPI with the conventional photomultiplier tube.

### 2.9 Amino acid analysis by GC–MS

GC–MS analysis of amino acid levels within either cell lysate or brain tissue was adapted from an earlier study (Svagera, Hanzlikova, Simek, & Husek, 2012), where 40 μl of sample (cell lysate or plasma) was spiked with 10 μl of DL‐4‐chlorophenylalanine (internal standard) followed by 10 μl of the reducing agent Tris(3‐hydroxypropyl)phosphine (0.5% w/v in water). Samples were gently mixed and left to stand for 1 min before adding 40‐μl trichloroacetic acid (0.6 M in water), vortexed twice, and samples centrifuged at 3,000 *g* for 10 min. Using 80 μl of supernatant, in a clean glass culture tube, 40 μl of a 3:1 1‐propanol:pyridine mixture was added, followed by 130 μl of a reactive mixture containing a 10:3:1 mix of 2,2,4‐trimethylpentane, butyl acetate, and propyl chloroformate. Samples were vortexed for 30 s and centrifuged at 3,000 *g* for 10 s, and the upper organic phase removed and used in GC–MS analysis. GC–MS was carried out on an HP5890 Series II chromatograph interfaced to an HP5972 MSD mass spectrometer; 2‐μl samples were injected in splitless mode (injector temp 240°C, purge delay 1.0 min) on a J&W DB5 column or Abel AB‐5MS (30 m × 0.25 mm dia × 0.25 μ film) using helium as the carrier gas at constant flow of 30 cm·s^−1^. The column oven was programmed from 90°C (2 min) to 300°C at 7.5°C·min^−1^, the GC–MS interface temperature was 300°C. Retention times and mass spectra of derivatives of amino acids were determined in TIC mode and confirmed to be in agreement with library and literature data. Quantitative analysis was carried out in SIM mode using 4‐chlorophenylalanine as internal standard. Chromatograms were integrated using MZmine (https://scicrunch.org/resources/Any/search?q=SCR_012040&l=SCR_012040) or manually using Agilent MSD Chemstation to yield peak areas of amino acid derivatives relative to that from the internal standard, and peak areas were processed using Prism software (https://scicrunch.org/resources/Any/search?q=SCR_005375&l=SCR_005375). To ensure unbiased analysis, samples were blinded and randomized prior to quantification. Samples size was estimated using data from a similar technique, where calculations with an observed effect of ≥20% suggested group size should be up to nine to observe an effect.

### 2.10 Treatment of *D*. *discoideum* with CBD for folate and amino acid (GC–MS and NMR) analysis

Cells (5 × 10^6^ cells) in the exponential phase of growth were plated onto a 10‐cm tissue culture dish in 10 ml of HL5 medium. Cells were allowed to adhere for 30 min, and then the medium was replaced containing 1.89‐μM CBD from 100‐mM stock in DMSO. Plates were maintained at 22°C for 12 hr. Following the 12‐hr incubation, cells were washed three times with KK2 buffer (16.2‐mM KH_2_PO_4_, 4‐mM K_2_HPO_4_), pelleted, and stored at −80°C. Prior to GC–MS analysis, 200 μl of sterile KK2 buffer with protease inhibitors was added to samples, which were briefly vortexed and subjected to five freeze thaw cycles from −80°C to 30°C. Samples were centrifuged at 5,000 *g* for 5 min and the cell lysate collected. The cell lysate was further centrifuged at 21,000 *g* for 10 min and the lysate collected for GC–MS analysis. To ensure unbiased analysis, samples were blinded and randomized prior to quantification.

### 2.11 Quantification of folate‐mediated one‐carbon metabolism intermediates by MS

Analysis of multiple folates was performed by UPLC–MS/MS as described previously (Leung et al., 2017; Pai et al., 2015). Buffer containing 20‐mM ammonia acetate, 0.1% ascorbic acid, 0.1% citric acid, and 100‐mM DTT at pH 7 was added to cell pellets. Cell suspensions were sonicated for 10 s using a hand‐held sonicator at 40% amplitude on ice. Protein was removed by precipitation with addition of two volumes of acetonitrile, mixing for 2 min and centrifugation for 15 min at 12,000 *g* and 4°C. Supernatants were transferred to fresh tubes, lyophilized, and stored at −80°C prior to analysis.

Lyophilized blinded samples were resuspended in 30‐μl water (Milli‐Q) and centrifuged for 5 min at 12,000 *g* at 4°C. Supernatants were transferred to glass sample vials for UPLC–MS/MS analysis. Metabolites were resolved by reversed‐phase chromatography using Acquity UPLC BEH C18 column (50 mm × 2.1 mm; 1.7‐μm bead size, Waters Corporation, UK). Solvents for UPLC were buffer A, 5% methanol, 95% Milli‐Q water, and 5‐mM dimethylhexylamine at pH 8.0; buffer B, 100% methanol. The column was equilibrated with 95% buffer A: 5% buffer B. The sample injection volume was 25 μl. The UPLC protocol consisted of 95% buffer A: 5% buffer B for 1 min, followed by a gradient of 5–60% buffer B over 9 min and then 100% buffer B for 6 min before re‐equilibration for 4 min. The metabolites were eluted at a flow rate of 500 nl·min^−1^. The UPLC was coupled to a XEVO‐TQS mass spectrometer (Waters Corporation, UK) operating in negative‐ion mode using the following settings: capillary 2.5 kV, source temperature 150°C, desolvation temperature 600°C, cone gas flow rate 150 L·hr^−1^, and desolvation gas flow rate 1,200 L·hr^−1^. Folates were measured by multiple reaction monitoring with optimized cone voltage and collision energy for precursor and product ions (as described in Leung et al., 2013).

### 2.12 LC–MS‐IT‐TOF analysis of cellular CBD levels in *D*. *discoideum*

Cell pellets, blinded and randomized prior to quantification, were homogenized in acetone and sonicated in an ultrasonic bath for 8 min. An internal standard for CBD quantification by isotope dilution (d4‐CBD 10 pmol) was added to the homogenate and extracted four times with acetone. The lipid‐containing solution was dried down, weighed, and pre‐purified by open bed chromatography on silica gel. Fractions were obtained by eluting the column with 99:1, 90:10, and 50:50 (v/v) chloroform/methanol. The 99:1 fraction was used for CBD quantification by LC–MS‐IT‐TOF analysis as described previously (Piscitelli, Pagano, Lauritano, Izzo, & Di Marzo, 2017).

### 2.13 Amino acid analysis by NMR

Intracellular metabolites were extracted as previously described (Viant, 2007). Briefly, a two‐phase extract was generated using a mixture of methanol, chloroform, and water in the volume ratio of 4:4:2.85. The aqueous phase containing water‐soluble low molecular weight endogenous metabolites was transferred to microtubes, and solvents were removed using a vacuum concentrator Eppendorf AG (Eppendorf, Hamburg, Germany), 8 hr at 60°C. Samples were then reconstituted in 70 μl of NMR phosphate buffer (pH 7.4) 0.2 M (80% of D_2_O, 20% of H_2_O, 3‐(trimethylsilyl)propionic‐2,2,3,3‐d4 acid; Sigma‐Aldrich) 1 mM, serving as NMR reference, vortexed for 10 s, and centrifuged at 12 000 g at 4°C for 10 min; 60 μl of resulting supernatant was pipetted into a 1.7‐mm capillary tube (Bruker, UK) for NMR analysis.

NMR analysis was carried out using a Bruker AV700 NMR instrument equipped with a 5‐mm inverse cryoprobe. A standard one‐dimensional nuclear Overhauser effect spectroscopy experiment was performed on each sample, using a standard preset pulse sequence (noesypr1d, 90° pulse length at 9.25 μs, total acquisition time of 2 s, and water pre‐saturation during relaxation delay of 5 s). All samples were analysed at 297 K and free induction decay was acquired on 19,607 data points (spectral width 9,803.9 Hz) using 512 scans (eight dummy scans). Free induction decay was then zero filled to 64 k points, and line broadening of 0.6 Hz was applied prior to fast Fourier transform. Phase and baseline corrections were performed manually using MestreNova software (version 10.0 m MestreLab Research). NMR spectra were referenced to 3‐(trimethylsilyl)propionic‐2,2,3,3‐d4 acid peak at 0 ppm. All samples were blinded prior to analysis.

### 2.14 Amino acid analysis of brain samples of a heterozygous *Scn1a* mouse model treated with CBD

129S‐*Scn1a* ^tm1Kea/Mmjax^ heterozygote (^+/−^) male mice (Jackson Laboratory, USA), maintained in the Bioresource Unit, University of Reading, were crossed with female C57BL/6 mice (Charles River, UK) to obtain hybrid *Scn1a* heterozygote (^+/−^) and wild‐type animals used in this study. This strain of *Scn1a* ^+/−^ mice show the characteristics of Dravet syndrome, a severe form of childhood epilepsy (Miller, Hawkins, McCollom, & Kearney, 2014). *Scn1a* ^+/−^ (epileptic) animals were randomly assigned to either vehicle (ethanol: kolliphor®: 0.9% saline = 2:1:17; *n* = 10) or CBD (100 mg·kg^−1^ twice daily subcutaneous injection, *n* = 10; GW Pharmaceuticals batch number 070214) for 6 weeks from postnatal Day 8 onwards. Likewise, a wild‐type group was taken as healthy control (*n* = 9) and treated with vehicle for 6 weeks. All the animals were group housed and maintained in a 12 hr:12 hr dark:light cycle at an RT of 21°C and humidity of 50 ± 10%, with ad libitum access to food and water. This experiment was conducted in the dark cycle (dim red light 8:00–20:00), and injections were made at circa 8 a.m. and circa 8 p.m. by both male and female experimenters. The dose of CBD used here was selected to compare with the effective clinical dose used in humans to treat Dravet syndrome and epilepsy in other rodent models (Patra et al., 2019) estimated using a method as described earlier (Nair & Jacob, 2016). At the end of the treatment period, all animals were humanely killed by cervical dislocation for collection of blood (heparinized tube; Fisher Scientific, UK) and brain (flash frozen in liquid nitrogen). Tissue was stored at −80°C until used in amino acid analyses. The experiments were conducted following UK Home Office regulations (Animals [Scientific Procedures] Act, 1986) under licence 70/8397 “Mouse Model of Dravet Syndrome” and was approved by the Animal Welfare and Ethics Review Board at the University of Reading. We used previous data from a similar technique and standard sample size calculations to estimate group size to observe an effect ≥20%, suggesting a group size should be up to nine animals to observe an effect. To ensure unbiased analysis, samples were blinded and randomized prior to quantification. Unfortunately, we could not determine the effect of CBD on one‐carbon cycle amino acids in healthy animals as our previous preliminary data did not warrant inclusion of this group in a chronic dosing study as CBD is well tolerated in healthy rodents. Animal studies are reported in compliance with the ARRIVE guidelines (Kilkenny, Browne, Cuthill, Emerson, & Altman, 2010) and with the recommendations made by the *British Journal of Pharmacology.*

### 2.15 Acute seizure model of rat primary hippocampal neurons treated with pentylenetetrazol and CBD

Pregnant female Sprague‐Drawley rats (4–5 months old) at 18 days of gestation were culled by concussion. The embryos were removed and 5 × 10^5^ cells were extracted from the embryo hippocampi, seeded onto a six‐well plate and matured for 21 days in modified neurobasal medium (Thermo Fisher Scientific, UK) with B27 supplement (1 ml/50 ml). Intervention groups were treated with CBD from 100‐mM stock in DMSO to a final concentration of 1.89 μM and incubated at 37°C and 5% CO_2_ with sample provided for analysis randomized and blinded. After 1 hr of incubation, both intervention and untreated cells were treated with pentylenetetrazol (PTZ) from 1‐M stock to a final concentration of 5 mM and placed at 37°C and 5% CO_2_ for 20‐min cells. Media was aspirated and the cells were washed three times with ice cold PBS. Cells were lysed for 10 min using 200‐μl RIPA buffer containing protease inhibitors and then collected using a cell scraper. Samples were immediately frozen at −80°C and used in subsequent GC–MS and protein analyses.

### 2.16 Data and analysis

The data and statistical analysis comply with the recommendations of the *British Journal of Pharmacology* on experimental design and analysis in pharmacology (Curtis et al., 2018). The distribution of all experimental data was tested for using the Anderson–Darling test for normality. All data that showed a Gaussian distribution were analysed using parametric tests. No outliers were excluded from analysis. Normally distributed data from two groups were statistically analysed using a Student's *t*‐test. The one‐way ANOVA statistical test was used to test for significance between the means of three or more independent groups of normally distributed data. In conjunction with the one‐way ANOVA, Tukey multiple comparison tests were performed to test for significance between all possible pairs of means. All experimental data involving two independent variables were analysed using a two‐way ANOVA. The two‐way ANOVA was used to test for an interaction between these independent variables and the dependent variable. In conjunction with the two‐way ANOVA, a Bonferroni multiple comparison test was performed to test for significance between all possible pairs of means. Post hoc analysis in ANOVA tests was only performed if the F‐test value of the ANOVA reached significance (*P* < .05). N values are defined as a sample derived from an individual animal or experiment, that is, technical replicates were not considered as an *n* value. Only data with *n* ≥ 5 were statistically analysed. Data were considered statistically significant if *P* < .05. Data provided in the results are not paired, and thus the number in each group does not need to be identical (providing each set of data was sufficiently powered). We have thus always performed a minimum of six experiments and that each group was statistically powered to see the effect size observed.

### 2.17 Materials

CBD was provided by GW Research Ltd.

### 2.18 Nomenclature of targets and ligands

Key protein targets and ligands in this article are hyperlinked to corresponding entries in http://www.guidetopharmacology.org, the common portal for data from the IUPHAR/BPS Guide to PHARMACOLOGY (Harding et al., 2018), and are permanently archived in the Concise Guide to PHARMACOLOGY 2017/18 (Alexander et al., 2017).

## 3 RESULTS

### 3.1 Investigating cellular mechanisms of CBD in the model system *D*. *discoideum*

**Figure 1:**
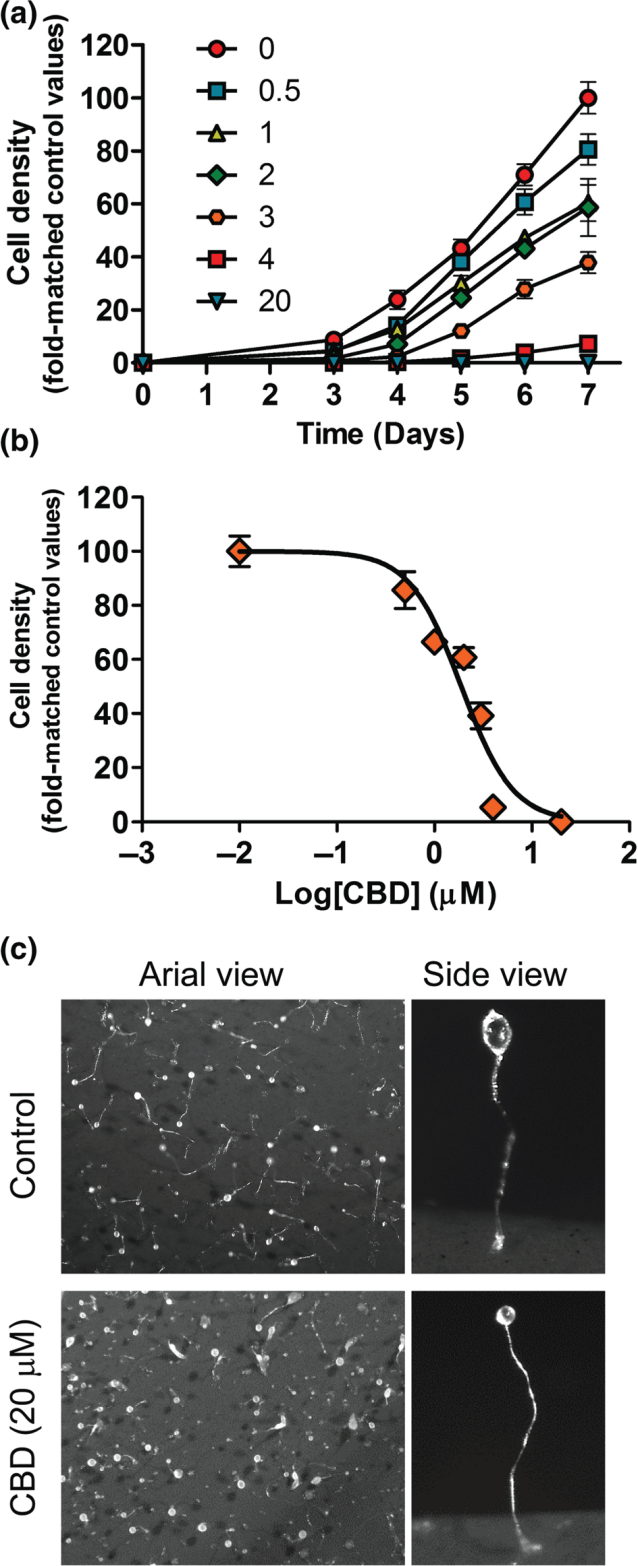
Cannabidiol (CBD) is likely to have molecular targets in *D*. *discoideum* cell affecting proliferation but not development. (a) The effect of CBD on *D*. *discoideum* cell growth was treated over a 7‐day period under a range of indicated concentrations (μM), with CBD blocking growth at 4 μM (data derived from 6 to 9 independent experiments) and presented as normalized to fold change from control values (untreated, Day 7). (b) A concentration–response curve of normalized cell density against the Log (concentration) of CBD was used to calculate the IC_50_ value with a 95% confidence interval. (c) The effect of CBD on *D*. *discoideum* development was analysed using cells plated upon nitrocellulose filters under starvation conditions where fruiting bodies were formed, shown for a field of fruiting bodies (aerial view) and for a single fruiting body. Treatment of cells with either 20 μM CBD or vehicle alone did not block development (*n* = 9)

To investigate a molecular mechanism of CBD, independent of previously described targets and effects, we initially employed the tractable model *D.* *discoideum.* Treatment with CBD at concentrations from 0 to 20 μM caused a concentration‐dependent inhibition of unicellular growth, providing a readout for mechanistic studies (Figure 1a). Growth inhibition had an IC_50_ of 1.9 μM (95% CI [1.6–2.2 μM]; Figure 1b), where growth was significantly reduced at 0.5 μM (*P* < .05) and blocked at 20 μM CBD. This concentration is close to steady‐state plasma levels shown in clinical studies (Devinsky et al., 2017; Devinsky et al., 2018), rather than high concentrations reported in some investigations (Bih et al., 2015). These data suggest the presence of CBD‐sensitive processes in *D.* *discoideum*, with regulation of a potential target at similar concentrations shown to block seizure activity in mammalian models (Jones et al., 2012; Kaplan et al., 2017; Klein et al., 2017).

We continued by analysing the effect of CBD on *D.* *discoideum* development, a process where starvation initiates aggregation and the formation of multicellular fruiting bodies over a 24‐hr period (Kelly et al., 2018). In this model*,* development and cellular growth can be mutually exclusive cell functions, controlled by groups of both independent and common proteins, providing the opportunity to assess the effect of CBD on both functions. Cell aggregation and development led to the formation of mature fruiting bodies in the absence and presence of CBD (20 μM; Figure 1c), demonstrating that CBD had no effect on development. Hence, the CBD target is necessary for cellular growth but is not involved in multicellular development, suggesting a specific molecular effect in this model rather than a general toxic effect.

### 3.2 Identification of *D*. *discoideum* GCVH1 and *H*. *sapiens* GCSH as regulators of CBD cellular function

**Figure 2:**
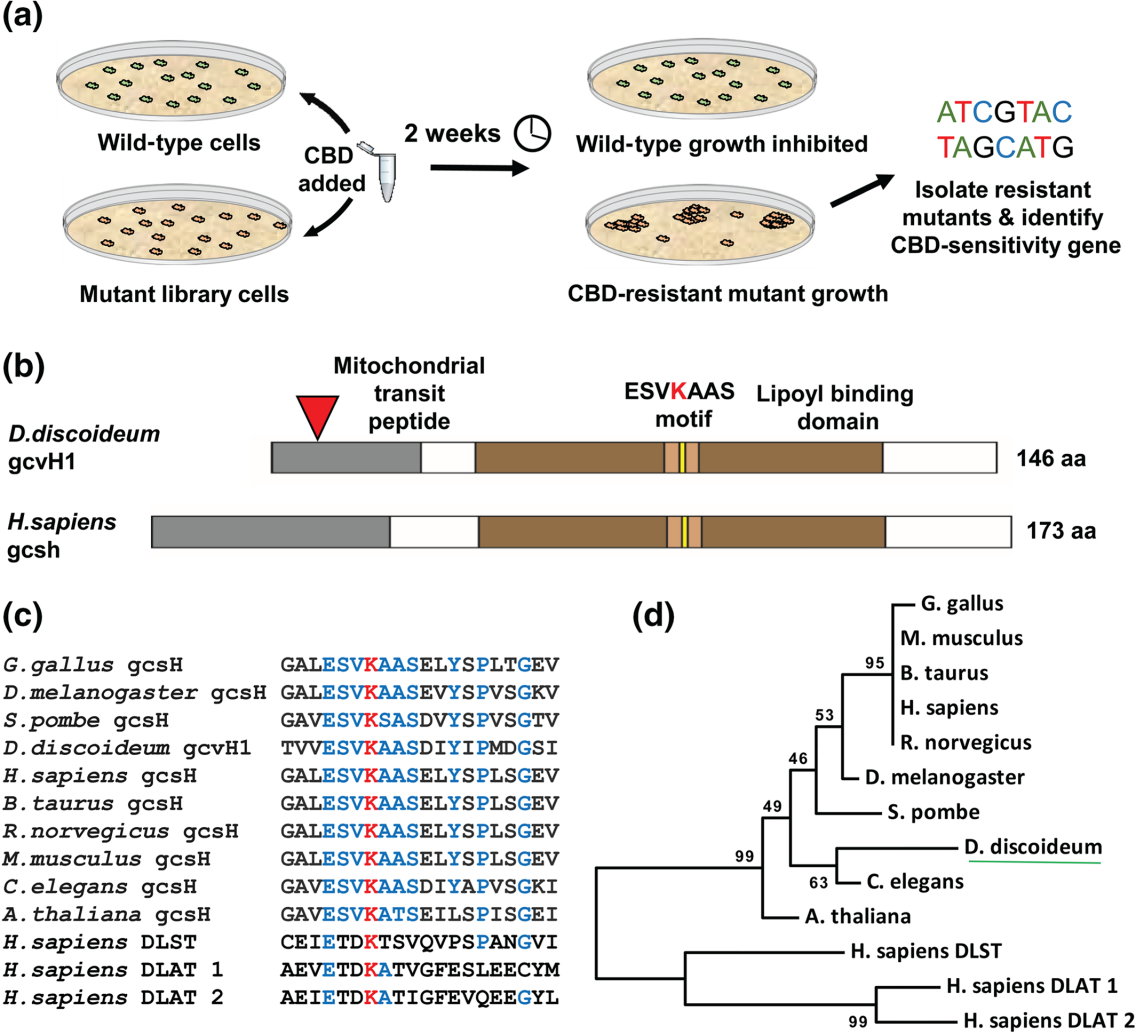
Identifying a role for the glycine cleavage system component, GCVH1, in regulating the cellular effect of CBD in *D*. *discoideum*. (a) A genetic screen of an insertional mutant library was carried out to identify mutants unaffected by the inhibitory effects of CBD on growth. (b) This screen identified GCVH1 as a potential molecular target for CBD, with an insertion in the first of three exons (red triangle), interrupting the coding region. Both the *D*. *discoideum* and the *H*. *sapiens* GCVH1 proteins are of similar size and contain highly conserved functional domains, include a mitochondrial localization sequence/transit peptide and a lipoyl binding domain, containing the ESVKAAS motif for lipoic acid binding. (c) The lipoic acid binding motif is highly conserved across a broad range of species but absent in the related dihydrolipoyllysine‐residue succinyltransferase (DLST/DLAT) proteins. (d) Such conservation highlights the evolutionary similarity of *D*. *discoideum* GCVH1 to orthologues found in higher organisms

In order to understand the effect of CBD on *D.* *discoideum* growth, we sought to identify molecular requirements for CBD action by isolating mutants with reduced CBD sensitivity in a genetic screen of an insertional mutant library (Figure 2a). A library of mutants was treated with CBD at a concentration that inhibits cell growth by 80% (9.5 μM). After 2‐week incubation, four CBD‐resistant mutants were isolated and the insertionally inactivated gene potentially controlling CBD sensitivity was identified using inverse PCR (Keim, Williams, & Harwood, 2004) or whole‐genome sequencing (Figure S1). This approach identified a CBD‐resistant *gcvH1* ^−^ mutant, with an insertion in exon 1 of *gcvH1* (DDB_G0287773) encoding the glycine cleavage system H protein (GCVH1; Figure 2b). The *gcvH1* ^−^ mutant was recapitulated in wild‐type cells by removal of a central part of the encoding gene by homologous recombination and the mutant was confirmed to have lost *gcvH1* expression (Figures S2 and S3).

We further assessed if *D.* *discoideum* GCVH1 is a likely homologue of GCSH (UniProt P23434). GCSH and GCVH1 are of similar size and domain structure and contain a mitochondrial‐targeting peptide sequence at the N‐terminus (Figure 2b). Similarly, both proteins possess a lipoyl‐binding domain containing a highly conserved motif (Figure 2c), including a key lysine residue that binds the lipoic acid co‐factor to facilitate shuttling of the glycine intermediates within the GCS. This motif is highly conserved throughout the kingdom of life (Figure 2d; Kikuchi & Hiraga, 1982). This analysis suggests that the *D.* *discoideum* GCVH1 protein is likely to represent a homologue of the mammalian GSCH, with similar catalytic activity and function. Since changes in glycine (Boison, 2016; Lynch, 2004; Xiong et al., 2012) and components of glycine in the one‐carbon cycle such as adenosine have been associated with epilepsy (Boison, 2016; Kobow et al., 2013), we continued our analysis of this mutant rather than the other identified mutants (Figure S1).

**Figure 3:**
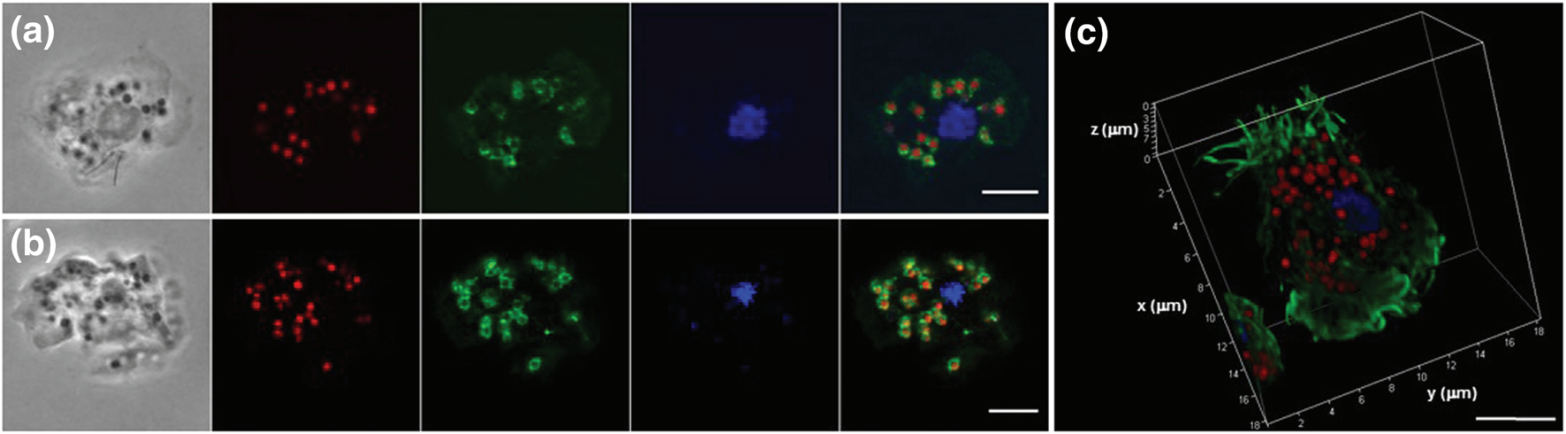
The *D*. *discoideum* GCVH1 and *H*. *sapiens* GCSH proteins localize to mitochondria in *D*. *discoideum*. (a) Confocal imaging of *D*. *discoideum* gcvH1^*−*^ cells expressing *D*. *discoideum* GCVH1‐RFP, imaged using phase contrast, with RFP fluorescence (red), a porin antibody (green), and DAPI to visualize DNA (blue), and with a merged fluorescence image on the right. (b) Shows *D*. *discoideum* gcvH1^*−*^ cells expressing *H*. *sapiens* GCSH‐RFP, imaged using phase contrast, with RFP fluorescence (red), a porin antibody (green), and DAPI to visualize DNA (blue), and with a merged fluorescence image on the right. (c) Three‐dimensional reconstruction of *D*. *discoideum* gcvH1^*−*^ cells expressing *D*. *discoideum* GCVH1‐RFP, using RFP fluorescence (red), filamentous actin imaged using Atto488‐phalloidin (green), and DAPI to visualize DNA (blue). Scale bars correspond to 5 μm

We then investigated whether the *D.* *discoideum* GCVH1 protein localizes to mitochondria, consistent with the localization of GCSH in mammalian systems using fluorescently tagged gcvH1 (GCVH‐RFP) expressed in *gcvH1* ^−^ cells (Figures 3 and S3). Visualization of the resulting GCVH1‐RFP expressing cells showed localization in small intracellular dots corresponding to mitochondria seen by phase‐contrast microscopy (Figures 3a, S4, S5, and S6). A similar localization was seen on overexpression of *H*. *sapiens* GCSH‐RFP (Figures 3b and S4). To validate this localization, cells were fixed and probed with antibodies to both RFP and porin, a mitochondrial protein (Troll et al., 1992). Porin labelling encapsulated both GCVH1‐RFP and GCSH‐RFP proteins (Figure 3a,b), consistent with a mitochondrial matrix localization of both GCS proteins and the mitochondrial membrane localization of porin. These data, together with three‐dimensional reconstruction of labelling (Figures 3c, S4, S5, and S6), confirm a localization of the GCVH1 and the *H*. *sapiens* orthologue GCSH in mitochondria in *D.* *discoideum*, consistent with a role for these proteins in the GCS.

**Figure 4:**
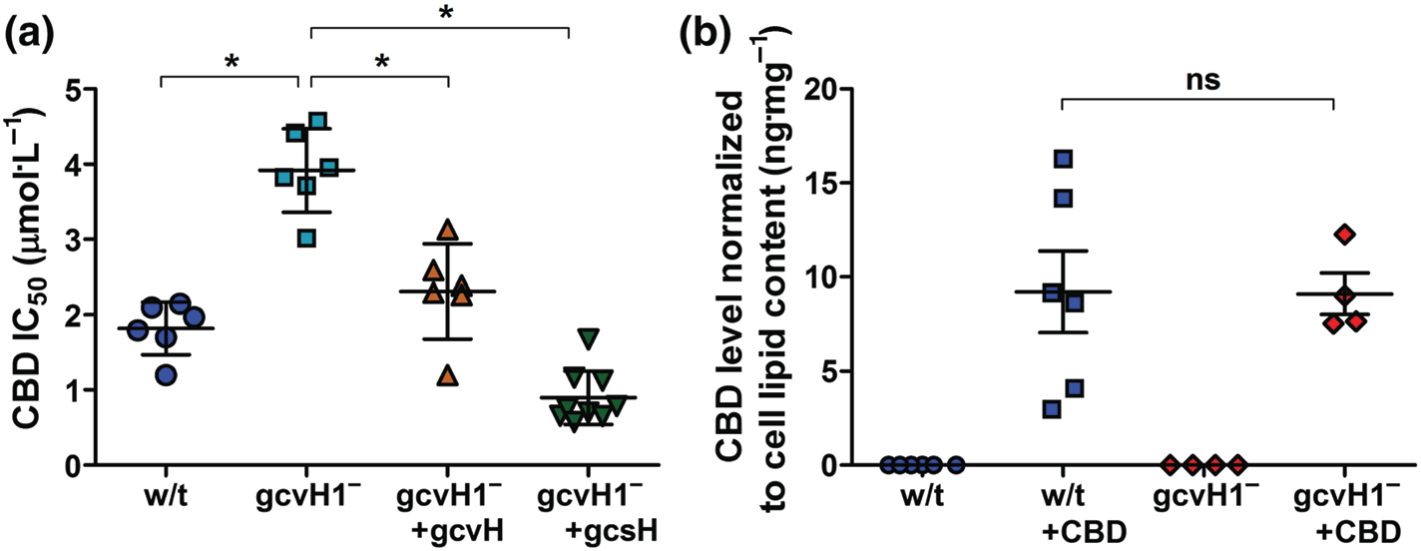
The CBD‐resistant phenotype through loss of GCVH1 is rescued by both *D*. *discoideum* GCVH1 and *H*. *sapiens* GCSH proteins and is not caused by reduced CBD uptake or increased removal/degradation. (a) Concentration–response assays of wild‐type, gcvH1^*−*^, and rescue cell lines with either the *D*. *discoideum* or *H*. *sapiens* genes replaced was carried out. Data are mean +/− SEM (^*^, *P* < .05 one‐way ANOVA, Tukey post hoc test). (b) Cellular CBD levels were quantified within wild‐type *D*. *discoideum* and gcvH1^*−*^ cell lines using LC–MS‐IT‐TOF following a prolonged CBD treatment regime (*n* = 4 or 6 as indicated). Data are mean +/− SEM (*P* > .05, Student's *t*‐test)

To confirm a role for gcvH1 in the regulation of CBD sensitivity, growth resistance to CBD was assessed in the *gcvH1* ^*−*^ mutant and following rescue with GCVH1‐RFP and GCSH‐RFP proteins. The *gcvH1* ^*−*^ cell line showed partial resistance to CBD (Figures 4a and S7) with a growth inhibitory constant (IC_50_) of 4.2 μM (95% CI [3.2–5.3 μM]). Expressing either *D.* *discoideum* GCVH1‐RFP or *H*. *sapiens* GCSH‐RFP protein in *gcvH1* ^*−*^ restored sensitivity to growth inhibition, with IC_50_ of 2.2 μM (95% CI [1.6–2.9 μM]) and 0.82 μM (95% CI [0.67–1.0 μM]) respectively (Figures 4a, S3, and S7). These data support a role for both GCVH1 and GCSH in regulating CBD sensitivity, in addition to confirming a common function of the *D.* *discoideum* and the *H*. *sapiens* proteins.

To ensure the resistance observed in the *gcvH1* ^−^ mutant was not from increased xenobiotic degradation or a decreased uptake and increased removal of CBD, we quantified cellular CBD levels in mutant and wild‐type cells. Here, cells were treated with CBD (1.89 μM) for 12 hr, and cellular CBD levels were quantified by LC–MS. No significant difference in CBD levels was found between wild‐type and the *gcvH1* ^−^ cell lines (Figure 4b). This supports the hypothesis that the effect of CBD is dependent upon a pathway controlled by the mitochondrial GCS (GCVH1 or GCSH) but not by CBD metabolic degradation or transport.

### 3.3 Monitoring one‐carbon cycle amino acids and folates in *D*. *discoideum* following CBD treatment

**Figure 5:**
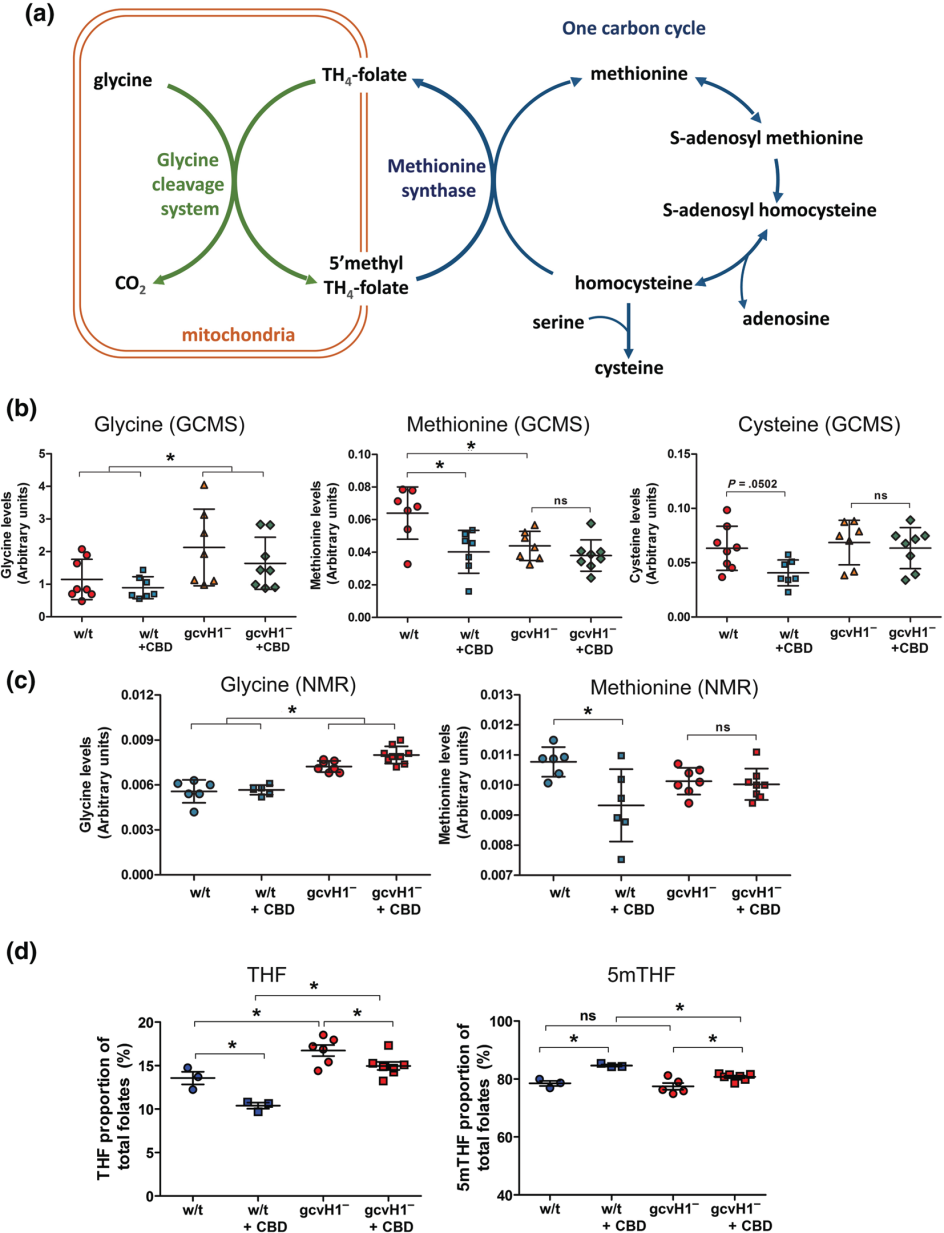
CBD treatment regulates the one‐carbon cycle and folate signalling dependent upon the glycine cleavage system in *D*. *discoideum*. (a) A schematic diagram showing how the glycine cleavage system (GCS—green) found within the mitochondria (orange) is linked to the one‐carbon cycle (blue), involving key amino acids—glycine, methionine, and cysteine, with methionine synthase providing the key point of interaction between the two systems. (b) To analyse changes in these systems, both wild‐type and gcvH1^*−*^ cell lines were treated with CBD (1.89 μM, 24 hr) or vehicle alone, and their amino acid composition was analysed by GC–MS (*n* = 6–9). Analysis shows that gcvH1 ablation and CBD treatment alters the levels of methionine and glycine. (c) Amino acid level changes were supported using NMR analysis (*n* = 6–9). All data are mean +/− SEM (^*^, *P* < .05, two‐way ANOVA, Bonferroni post hoc test). (d) Folate‐containing compounds tetrahydrofolate (THF) and 5‐methyltetrahydrofolate (5mTHF) were also assessed following equivalent treatment conditions (*n* = 3–7) consistent with a reduction in the glycine cleavage system by loss of GCVH1, and a CBD dependent increase in 5′mTHF consistent with a mechanism of CBD through inhibition of methionine production. All data are mean +/− SEM (^*^, *P* < .05, two‐way ANOVA, Bonferroni post hoc test)

We then sought to examine the molecular mechanism underlying the role of GCVH1 in CBD sensitivity, focusing on the function of the GCS and the interacting FOCM components (Figure 5a). In these experiments, we first examined the role of GCVH1 in regulating glycine, methionine, and cysteine levels using GC–MS. We found that glycine levels were significantly increased in *gcvH1* ^*−*^ cells compared to wild‐type cells (*P* < .05), with a moderate decrease in methionine levels (*P* < .05) and no change in cysteine levels, supporting a role for the encoded protein in the GCS (Figure 5b). We then examined the effect of CBD treatment in both wild‐type *gcvH1* ^−^ cells (using 1.89 μM for 12 hr), where CBD gave no change in glycine levels. In contrast, methionine levels were significantly reduced in wild‐type cells following CBD treatment (*P* < .05), and this effect was lost in *gcvH1* ^*−*^ cells, consistent with a CBD‐dependent inhibitory effect on methionine synthesis that is suppressed in *gcvH1* ^*−*^ cells (Figure 5b). Similar notable trend towards reduced cysteine levels (*P* = .0502) were observed in wild‐type cells following CBD treatment that was again absent in the *gcvH1* ^*−*^ mutant (Figure 5b). These data support a role for CBD in reducing methionine synthase activity, dependent upon GCS activity. We independently confirmed these changes using an NMR‐based approach with an identical treatment regimen used in the GC–MS analysis. Again, glycine levels were elevated in the *gcvH1* ^−^ mutant compared to wild‐type cells (*P* < .05; Figure 5c) but did not change following CBD treatment. Here, CBD treatment again caused a significant reduction in wild‐type methionine levels (*P* < .05) with this effect lost in the *gcvH1* ^−^ mutant (Figure 5c), again consistent with GC–MS results. Thus, these two distinct approaches highlight a common effect of CBD in reducing methionine levels and show that the effects of CBD on these components are reliant upon activity of the GCS.

We continued by analysing the potential effect of CBD treatment on the relative abundance of folate mediators of one‐carbon metabolism, with exploratory analysis of wild‐type and *gcvH1* ^−^ cells, using LC–MS/MS. Comparison of folate profile in wild‐type and untreated *gcvH1* ^−^ cells identified a significant (*P* < .05) increase in tetrahydrofolate (THF) levels in mutant, consistent with impaired activity of the GCS (Leung et al., 2017; Figure 5d). Following CBD treatment, wild‐type cells showed a significant 23% reduction in THF levels (*P* < .05), with a concomitant 8% increase in 5‐methylTHF (5mTHF; *P* < .05; Figure 5d). As with lower methionine abundance, this effect is consistent with a CBD‐dependent block in methionine synthase activity. In comparison, *gcvH1* ^−^ cells showed a reduced sensitivity to the effect of CBD, with only a 7% increase in THF and a 3% decrease in 5mTHF following treatment (both *P* < .05). Hence, difference in THF levels between *gcvH1* ^−^ cells and wild‐type cells (*P* < .05) was of greater magnitude following CBD treatment (Figure 5d). These data implicate a role for CBD in reducing methionine synthase activity in this model as a potential mechanism underlying diminished THF production, with ablation of *gcvH1* ^−^ attenuating this effect.

### 3.4 Monitoring amino acid regulation in a Dravet syndrome epilepsy model following CBD treatment

**Figure 6:**
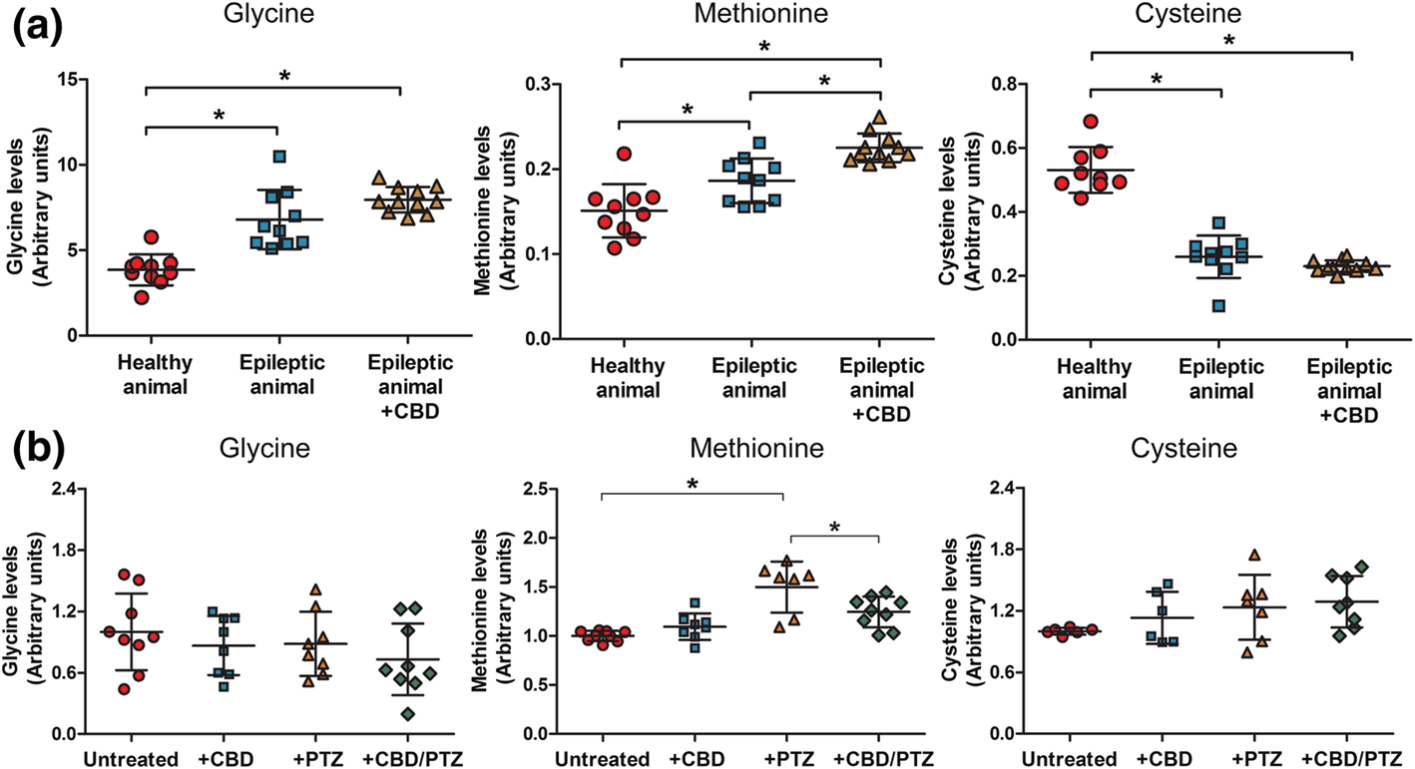
A Dravet syndrome model and an in vitro seizure model implicate the one‐carbon (amino acid) cycle as a potential mechanistic target of cannabidiol (CBD) treatment in epilepsy. (a) Analysis of one‐carbon cycle amino acids (glycine, methionine, and cysteine) in a mouse *Scn1a* ^*+/−*^ Dravet model suggests significant dysregulation of these amino acids in brain tissue, with chronic CBD‐treatment further modifying methionine levels. (b) Analysis of rat primary hippocampal neurons, pretreated with CBD (60 min 1.89 μM), or following induction of seizure‐like activity induced with PTZ (with or without CBD treatment), shows no alteration in glycine or cysteine levels but demonstrates elevates methionine levels upon seizure induction, with this increase attenuated by CBD treatment. Data are shown as mean ± SEM (^*^, *P* < .05, one‐way ANOVA, Tukey post hoc test, *n* ≥ 7)

We then investigated potential changes in amino acid signalling in epilepsy models. First, we employed a heterozygous *Scn1a* ^*+/−*^ mouse model lacking one copy of the Na_V_1.1 protein (Thiele et al., 2018) that is used as a model for a variety of seizure types from simple febrile seizures to severe genetic disorders such as Dravet (Nakayama et al., 2018) and Lennox–Gastaut syndromes (Zhou et al., 2018). We initially analysed brain amino acid levels in healthy (wild‐type) and *Scn1a* ^*+/−*^ mice, where a significant increase in glycine (*P* < .05) and methionine (*P* < .05) levels and a decrease in cysteine (*P* < .05) levels were found in these epileptic model mice compared to wild‐type controls (Figure 6a). We also examined potential changes in these amino acids following 6‐week CBD treatment (twice daily subcutaneous injection at 100 mg·kg^−1^). In CBD‐treated *Scn1a* ^*+/−*^ animals, glycine, methionine, and cysteine remained deregulated compared to untreated animals (Figure 6a), consistent with a role of these elevated amino acid levels providing a trait marker for Dravet syndrome, although methionine levels significantly increased (*P* < .05) following CBD treatment above that of untreated animals. This CBD‐dependent increase was unexpected, as brain levels of methionine are normally tightly regulated in healthy animals (Gupta et al., 2004; Selmer, Lund, Brandal, Undlien, & Brodtkorb, 2009). These insights suggest that in the Dravet model, extended treatment with CBD modulates levels of methionine, a key component of one‐carbon metabolism.

### 3.5 Monitoring one‐carbon metabolism amino acids in an in vitro seizure model following CBD treatment

Since the Dravet model mice showed altered levels of methionine following long‐term CBD treatment, we also employed an in vitro seizure model to investigate potential changes in this amino acid (and glycine and cysteine) at a cellular level following acute CBD treatment. Here, primary hippocampal neurons from wild‐type rats were matured for 21 days to develop synaptic connections and pretreated with CBD (1.89 μM for 60 min) followed by the induction of seizure‐like activity by treatment with 5‐mM PTZ for 20 min (Chang et al., 2014). PTZ is known to be a GABA_A_ receptor antagonist and has been used extensively in epilepsy research to induce seizure activity by preventing the inhibitory response of GABA (Armand, Louvel, Pumain, & Heinemann, 1998). In this acute model, neurons showed a significant increase in methionine levels following seizure induction (*P* < .05), consistent with that observed in the Dravet model brain, while this increase was significantly reduced by pretreatment with CBD (*P* < .05; Figure 6b). No significant changes in cysteine or glycine levels were found following seizure‐like activity or with CBD treatment in this model (Figure 6b). These data suggest that induction of seizure‐like activity elevates neuronal methionine levels, and acute CBD treatment attenuates this effect. Thus, both chronic and acute CBD treatment regulate methionine levels, suggesting a novel effect not previously reported and potentially related to the therapeutic mechanism of CBD.

## 4 DISCUSSION

Understanding the therapeutic mechanism(s) of *Cannabis*‐derived compounds has been a recent priority in research, particularly in the treatment of epilepsy. Here, we investigated potential mechanisms underlying the molecular function of CBD using *D.* *discoideum* as a tractable 3Rs (replacement, refinement, or reduction) model organism that has been used in a range of other pharmacogenetic studies including those focused on epilepsy (Chang et al., 2012; Kelly et al., 2018; Warren et al., 2018). Using this model enabled the identification of a mechanism for CBD dependent upon the mitochondrial GCS protein, GCVH1, a component of FOCM, where this role that is conserved with the human orthologue (GCSH). We demonstrated that GCVH1 localized to mitochondria, consistent with studies of GCSH in other models (Lamers et al., 2009; Leung et al., 2017). Loss of GCVH1 significantly elevated glycine levels, increased THF levels, and reduced 5mTHF levels, all confirming impaired activity of the GCS in this mutant (Figure 5). Loss of GCVH1 also led to lower methionine levels (using GC/MS analysis), consistent with impaired GCS activity leading to decreased conversion of homocysteine to methionine via methionine synthase (https://www.guidetopharmacology.org/GRAC/ObjectDisplayForward?objectId=3099). Thus, loss of GCVH1 gives rise to metabolic outcomes that resembles those in mouse and human studies (Lamers et al., 2009; Leung et al., 2017) and highlights an evolutionarily conserved function of this important metabolic pathway.

We then demonstrated that CBD functions to modulate methionine levels and folate intermediates in the one‐carbon cycle, dependent upon GCVH1 activity, revealing a novel role for CBD in inhibition of methionine production. The increased resistance of *gcvH1* ^*−*^ mutants to CBD is modest, with a twofold decrease in sensitivity, suggesting that this mechanism may represent one of multiple cellular mechanisms commonly found in therapeutic natural products and drugs (Kobayashi, Endoh, Ohmori, & Akiyama, 2019; Zhang, Huai, Miao, Qian, & Wang, 2019) but still demonstrates a role for the GCS in regulating CBD cellular effects. However, in our studies, CBD treatment did not increase glycine levels, arguing against a direct inhibitory effect of CBD on the GCS. Excitingly, treatment of wild‐type cells with CBD lowered methionine levels, and this effect was lost in the *gcvH1* ^−^ mutant, suggesting a mechanism of CBD action through inhibition of methionine production. Although we were unable to detect homocysteine in cells, cysteine levels showed a trend for a CBD‐dependent reduction in wild‐type cells that was absent in the *gcvH1* ^−^ mutant, also consistent with a role for CBD in regulating GCS‐dependent one‐carbon metabolism. CBD treatment also caused a decrease in relative abundance of THF and an increase in 5mTHF, supporting the hypothesis of a role for CBD in suppressing methionine synthase activity, with this affect attenuated by loss of GCVH1. These data therefore provide evidence for CBD‐dependent regulation of one‐carbon metabolism in *D.* *discoideum*, in which methionine levels are lowered in a GCS‐dependent manner, revealing a new molecular pathway modulated by CBD.

We then sought to translate this research to a relevant epilepsy model. Employing a mouse *Scn1a* ^*+/−*^ mutant to investigate a potential mechanism of CBD provided a logical approach, as this model reproduces seizure types found in patients with Dravet syndrome (Marini et al., 2011) and Lennox–Gastaut syndrome (Selmer et al., 2009). Surprisingly, we demonstrated that brain levels of glycine, methionine, and cysteine were all significantly altered in the model, suggesting that neuronal levels of these amino acids may provide trait markers for epilepsy. Interestingly, epilepsy is a key feature of non‐ketotic hyperglycinemia (also known as glycine encephalopathy), an autosomal recessive neurometabolic disorder characterized by elevated levels of glycine in blood and CSF (Swanson et al., 2015) caused by mutation of components of the GCS (Kure et al., 2006). These observations provide a potential link between Dravet syndrome, mitochondrial function and glycine levels, and non‐ketotic hyperglycinemia. Since mitochondrial dysfunction in Dravet syndrome has been suggested from studies of patient skin fibroblasts (Doccini et al., 2015), in patients (Panneman et al., 2018), in a zebrafish model (Kumar et al., 2016), and in epilepsy‐related oxidative stress (Pearson‐Smith et al., 2017), our data support and extend these findings. Thus, this mechanism may now be added to those already proposed for CBD in epilepsy treatment (Xiong et al., 2012; Kaplan et al., 2017, Rodriguez‐Munoz et al., 2018, Vilela et al., 2017, Rimmerman et al., 2013).

In vivo studies can be complicated by chronic treatment causing long‐term cellular and physiological changes in the brain and the presence of a variety of cell types. To overcome these considerations, we analysed a role for CBD in regulating glycine, methionine, and cysteine levels using an acute neuronal seizure model (Chang et al., 2014). This approach defines acute changes found during seizures, and in response to drug treatment, where changes in level of these components would identify a role in seizures. We showed that seizure‐like activity did not alter glycine or cysteine levels in neurons themselves, however methionine levels were significantly increased, and this increase was attenuated by CBD treatment. These data are consistent with an acute role for CBD in reducing methionine production in seizure activity but do not prove this—since CBD may simply block seizure activity, and hence a seizure‐dependent increase in methionine. This could be examined in further experiments, with other anticonvulsants used to block seizure activity and methionine levels examined, and this represents a limitation in the present study. However, our results clearly indicate that seizure activity elevates methionine levels, revealing a new and unexpected effect of seizure activity. Differing effects of CBD treatment in the Dravet in vivo model and in primary neurons in vitro may be due to a range of factors including long‐term cellular and physiological changes in brain following treatment. Whole‐brain material also comprises a variety of cell types, where the GCS is mainly in glia cells not neurons, and these aspects will need further investigation.

One‐carbon metabolism includes a wide range of components implicated in neuronal excitability, seizure activity, and epilepsy. Glycine acts as a co‐agonist at excitatory NMDA receptors to provide a key neuronal glutamatergic (excitability) mechanism and through specific excitatory and inhibitory receptors (Lynch, 2004). The production of SAM provides a central function in the cycle, acting as the primary donor of methyl groups within cells for both DNA and neurotransmitter methylation (Moore et al., 2013). Adenosine has also been shown to regulate seizure activity (Boison, 2016) and is involved in DNA methylation to regulate epileptogenesis (Kobow et al., 2013), although CBD treatment attenuates the reduction in DNA methylation induced by cell differentiation (Pucci et al., 2013). Finally, homocysteine is a pro‐convulsant and is elevated in patients with recurrent seizure activity during treatment with antiepileptic drugs (Baldelli et al., 2010). Thus, one‐carbon metabolism provides a wide variety of potential impacts on seizure activity and epilepsy control, further emphasized by the current study.

In summary, our data provide new insights to altered metabolism in epilepsy as a disease state, and potential therapeutically relevant mechanisms of CBD action. Future research should thus investigate changes in one‐carbon metabolism in epilepsy, including Dravet syndrome, and other CBD‐treatable disorders, particularly where potential mitochondrial dysfunction may underlie some pathological symptoms. We further suggest that pharmacological regulation of methionine levels during seizure activity may provide a novel therapeutic approach for seizure control and one‐carbon cycle components may provide diagnostic markers within a clinical setting.

## CONFLICT OF INTEREST

B.J.W. is a current employee of GW Pharmaceuticals. The work was supported by GW Research Ltd.

## AUTHOR CONTRIBUTIONS

C.J.P., B.J.W., and R.S.B.W. designed the research. C.J.P., P.F., A.M.T., K.Y.L., E.W., J.O., D.S., P.H.P., S.G., J.B., F.P., R.J.H.,S.P.C., J.B., A.M., and C.M.W. performed the research. B.S., A.B., A.H., C.T., and N.D.E.G. contributed new reagents or analytic tools. C.J.P., S.C., K.Y.L., R.S.B.W. analysed the data. C.J.P., R.J.H., N.D.E.G., and R.S.B.W. wrote the manuscript.

## DECLARATION OF TRANSPARENCY AND SCIENTIFIC RIGOUR

This Declaration acknowledges that this paper adheres to the principles for transparent reporting and scientific rigour of preclinical research as stated in the *BJP* guidelines for https://bpspubs.onlinelibrary.wiley.com/doi/full/10.1111/bph.14207, https://bpspubs.onlinelibrary.wiley.com/doi/full/10.1111/bph.14208, and https://bpspubs.onlinelibrary.wiley.com/doi/full/10.1111/bph.14206, and as recommended by funding agencies, publishers and other organisations engaged with supporting research.

## Supporting information


**Figure S1.** **Mutants identified in CBD‐resistance screen in *D. discoideum*.** Four loci were identified, including gcvH1, where the gene identifier (http://Dictybase.org) are provided, along with the encoded protein name, potential cellular roles, mutagenic insertion site, and number of independent mutants identified in the screen.
**Figure S2.** Primer combinations used for PCR amplification of *gcvH1* knockout cassette, mutant screening and cDNA cloning.
**Figure S3.** ***gcvH1* gene expression and protein presence was verified in the wild‐type, gcvH1‐ and rescue cell lines.** (A) The absence of GCVH1 in the null cell line (*gcvH1*‐), and the presence of either GCVH1 or GCSH mRNA in the rescue cell lines was confirmed by RT‐PCR. (B) The presence of either GCVH1‐RFP and GCSH‐RFP fusion proteins within the rescue cell lines was confirmed by Western blot analysis showing cropped bands and the entire gel.
**Figure S4.** **Confocal images of *D. discoideum* GCVH1 and *H. sapiens* GCSH localisation in *gcvH1*‐ cells.** Confocal imaging of *D. discoideum gcvH1*‐ cells expressing *D. discoideum* GCVH1‐RFP (DGcvH1 × 2) or human GCSH (HGCSH) imaged showing phase contrast, RFP fluorescence (red), Atto488‐phalloidin for filamentous actin (green), and DAPI for visualisation of DNA (blue). The merged fluorescence image is shown on the right. Images were deconvoluted using the Huygens software package (Scientific Volume Imaging). Scale bars correspond to 5 μm.
**Figure S5.** **Three‐dimensional reconstruction *of D. discoideum* GcvH1 localisation in *gcvH1*‐ cells.** GCVH1 is visualised by RFP fluorescence (red), with Atto488‐phalloidin for filamentous actin (green), and DAPI to visualize DNA (blue) in a merged fluorescent image that was deconvoluted using the Huygens software package. See also attached movie.
**Figure S6.** **Three‐dimensional reconstruction *of D. discoideum* GcvH1 localisation in *gcvH1*‐ cells.** GCVH1 is visualised by RFP fluorescence (red), with porin to visualise mitochondrial membrane (green), and DAPI to visualize DNA (blue) in a merged fluorescent image that was deconvoluted using the Huygens software package. See also attached movie. Page 37 of 36
**Figure S7.** **Dose‐response curves for *gcvH1‐* and rescue cell lines.** Comparison of CBD growth sensitivity of *D. discoideum* wild‐type (orange diamond), *gcvH1*‐ (red triangle), *gcvH1*‐::gcvH1‐RFP (blue circle), and *gcvH1*‐::gcsH‐RFP (green circle) cell lines. (A) Doseresponse curves of normalised cell density against the Log (concentration) for CBD was used to calculate the IC50 values with 95% confidence intervals for both the *gcvH1‐* and the rescue cell lines (*n* = 6–9, experiments were repeated).

Supporting info item

Supporting info item
