# Supplementary material for: A new mechanism for cannabidiol in regulating the one‐carbon cycle and methionine levels in Dictyostelium and in mammalian epilepsy models
Source: Br J Pharmacol. 2020 Jan 3;177(4):912–28. doi: 10.1111/bph.14892 (PMC7024701; doi:10.1111/bph.14892)
Supplement: Supplementary file 1 — Figure S1. Mutants identified in CBD‐resistance screen in D. discoideum. Four loci were identified, including gcvH1, where the gene identifier (http://Dictybase.org) are provided, along with the encoded protein name, potential cellular roles, mutagenic insertion site, and number of independent mutants identified in the screen. Figure S2. Primer combinations used for PCR amplification of gcvH1 knockout cassette, mutant screening and cDNA cloning. Figure S3. gcvH1 gene expression and protein presence was verified in the wild‐type, gcvH1‐ and rescue cell lines. (A) The absence of GCVH1 in the null cell line (gcvH1‐), and the presence of either GCVH1 or GCSH mRNA in the rescue cell lines was confirmed by RT‐PCR. (B) The presence of either GCVH1‐RFP and GCSH‐RFP fusion proteins within the rescue cell lines was confirmed by Western blot analysis showing cropped bands and the entire gel. Figure S4. Confocal images of D. discoideum GCVH1 and H. sapiens GCSH localisation in gcvH1‐ cells. Confocal imaging of D. discoideum gcvH1‐ cells expressing D. discoideum GCVH1‐RFP (DGcvH1 × 2) or human GCSH (HGCSH) imaged showing phase contrast, RFP fluorescence (red), Atto488‐phalloidin for filamentous actin (green), and DAPI for visualisation of DNA (blue). The merged fluorescence image is shown on the right. Images were deconvoluted using the Huygens software package (Scientific Volume Imaging). Scale bars correspond to 5 μm. Figure S5. Three‐dimensional reconstruction of D. discoideum GcvH1 localisation in gcvH1‐ cells. GCVH1 is visualised by RFP fluorescence (red), with Atto488‐phalloidin for filamentous actin (green), and DAPI to visualize DNA (blue) in a merged fluorescent image that was deconvoluted using the Huygens software package. See also attached movie. Figure S6. Three‐dimensional reconstruction of D. discoideum GcvH1 localisation in gcvH1‐ cells. GCVH1 is visualised by RFP fluorescence (red), with porin to visualise mitochondrial membrane (green), and DAPI to visualize DN [file BPH-177-912-s003.docx]

**Supplementary Figures Perry et al**

| ***Dictyostelium* gene identifier** | **Protein name** | **Potential cellular role** | **Insertion site** | **Number of independent mutants** |
| --- | --- | --- | --- | --- |
| DDB_G028773 | GcvH1 | Glycine Cleavage System | Exon 1 | 1 |
| DDB_G0281669 | Lmbd2b | Cobalamin transporter | Exon 2 | 12 |
| DDB_G0293904 | Reductase A (RedA) | Oxidoreductase enzyme, possible ancestor of cytochrome P450 or methionine synthase reductase | Upstream of start codon (185 bp) | 4 |
| DDB_G0288103 | SibB | Integrin-like protein, cell adhesion | Exon 3 | 3 |

**Figure S1. Mutants identified in CBD-resistance screen in *D. discoideum*.** Four loci were identified, including gcvH1, where the gene identifier (Dictybase.org) are provided, along with the encoded protein name, potential cellular roles, mutagenic insertion site, and number of independent mutants identified in the screen.

| 5’ KO targeting primers (281bp region) |  | ATAACTAGTGCCAAAAGAAACTTTTGTACTAGATAC |
| --- | --- | --- |
|  |  | ATACTGCAGCTGATTCAACTACTGTTATTGGTTG |
| 3’ KO targeting primers (343bp region) |  | ATACCATGGCGATATCTATATTCCAATGGATGG |
|  |  | ATAGGTACCCTTTGGAATTGATCAGTTTTGC |
| 5’ KO screening primers | Set 1 | CTTAAGATTTGGAACAAGAGCATTC |
|  | Set 1 | ATACTGCAGCTGATTCAACTACTGTTATTGGTTG |
|  | Set 2 | ATAACTAGTGCCAAAAGAAACTTTTGTACTAGATAC |
|  | Set 2 | AAAAAGATAAAGCTGACCCGAAAG |
|  | Set 3 | CTTAAGATTTGGAACAAGAGCATTC |
|  | Set 3 | AAAAAGATAAAGCTGACCCGAAAG |
| 3’ KO screening primers | Set 1 | TCAAATAATAATTAACCAACCCAAG |
|  | Set 1 | ATAGGTACCCTTTGGAATTGATCAGTTTTGC |
|  | Set 2 | ATACCATGGCGATATCTATATTCCAATGGATGG |
|  | Set 2 | CTTTAATATATTCATCATATTGGGC |
|  | Set 3 | TCAAATAATAATTAACCAACCCAAG |
|  | Set 3 | CTTTAATATATTCATCATATTGGGC |
| *gcvH1* cDNA cloning |  | ATAGAATTCATGTTAAAAACCTTAAGATTTGGAAC |
|  |  | ATAGGATCCATGTTCTTTAATATATTCATCATATTGG |

**Figure S2. Primer combinations used for PCR amplification of *gcvH1* knockout cassette, mutant screening and cDNA cloning.**


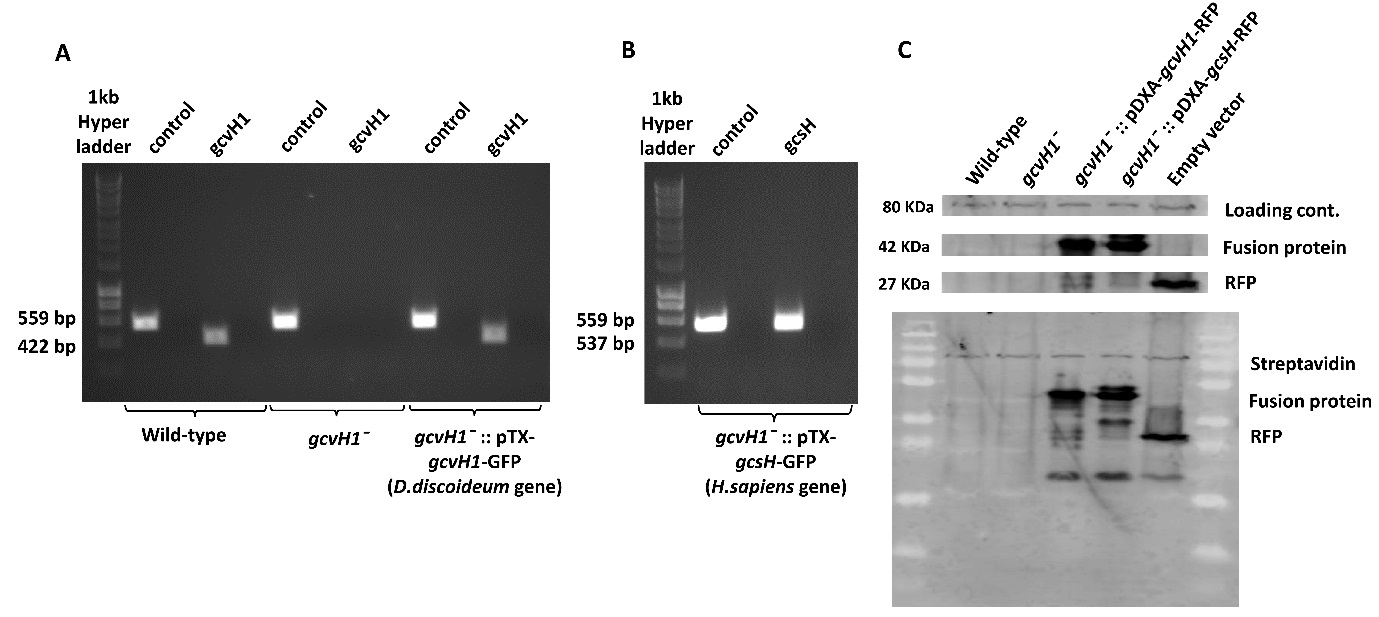


**Figure S3.  *gcvH1* gene expression and protein presence was verified in the wild-type, gcvH1^-^ and rescue cell lines.**  (A) The absence of GCVH1 in the null cell line (*gcvH1*^-^), and the presence of either GCVH1 or GCSH mRNA in the rescue cell lines was confirmed by RT-PCR. (B) The presence of either GCVH1-RFP and GCSH-RFP fusion proteins within the rescue cell lines was confirmed by Western blot analysis showing cropped bands and the entire gel.


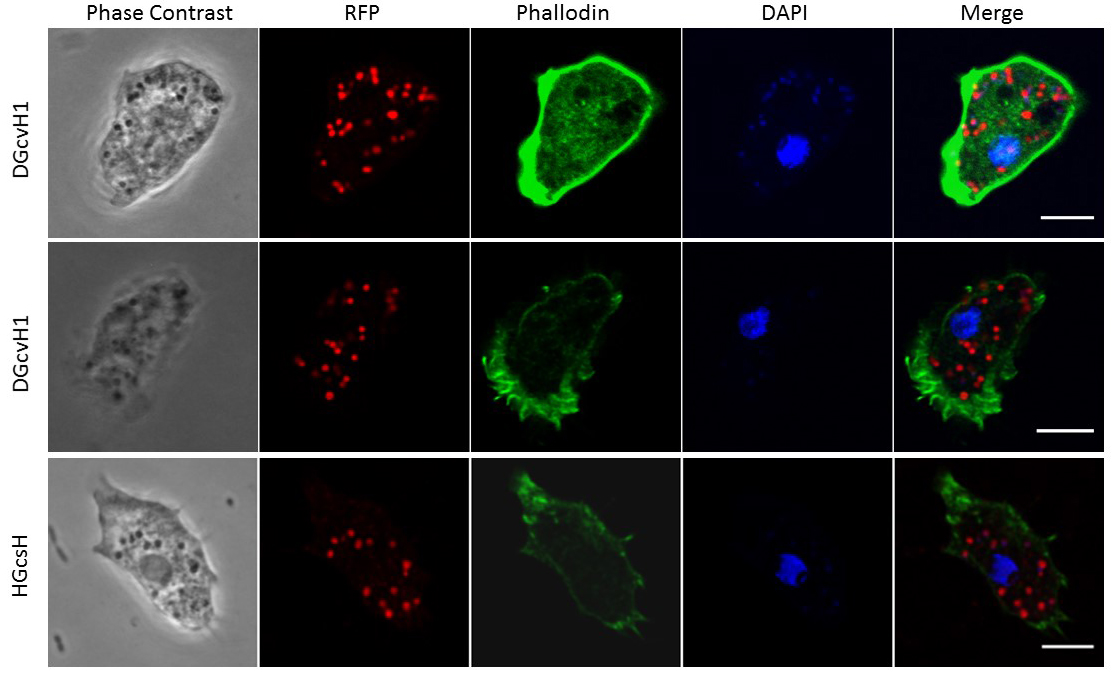


**Figure S4. Confocal images of *D. discoideum* GCVH1 and *H. sapiens* GCSH localisation in *gcvH1*^-^ cells.** Confocal imaging of *D. discoideum* *gcvH1*^-^ cells expressing *D. discoideum* GCVH1-RFP (DGcvH1 x 2) or human GCSH (HGCSH) imaged showing phase contrast, RFP fluorescence (red), Atto488-phalloidin for filamentous actin (green), and DAPI for visualisation of DNA (blue). The merged fluorescence image is shown on the right. Images were deconvoluted using the Huygens software package (Scientific Volume Imaging). Scale bars correspond to 5 µm.


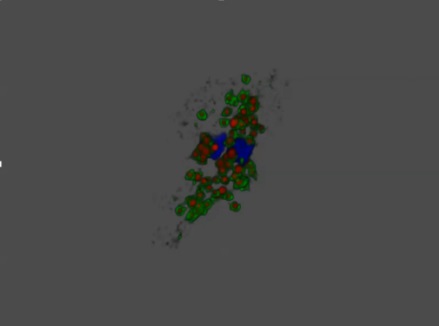

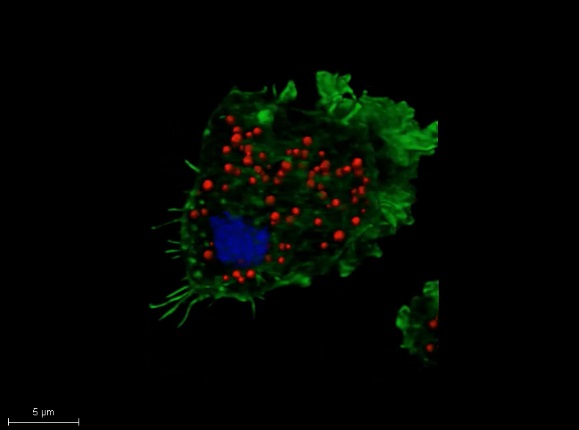
**Figure S5. Three-dimensional reconstruction *of D. discoideum* GcvH1 localisation in *gcvH1*^-^ cells.** GCVH1 is visualised by RFP fluorescence (red), with Atto488-phalloidin for filamentous actin (green), and DAPI to visualize DNA (blue) in a merged fluorescent image that was deconvoluted using the Huygens software package. See also attached movie.

**Figure S6. Three-dimensional reconstruction *of D. discoideum* GcvH1 localisation in *gcvH1*^-^ cells.** GCVH1 is visualised by RFP fluorescence (red), with porin to visualise mitochondrial membrane (green), and DAPI to visualize DNA (blue) in a merged fluorescent image that was deconvoluted using the Huygens software package. See also attached movie.

**
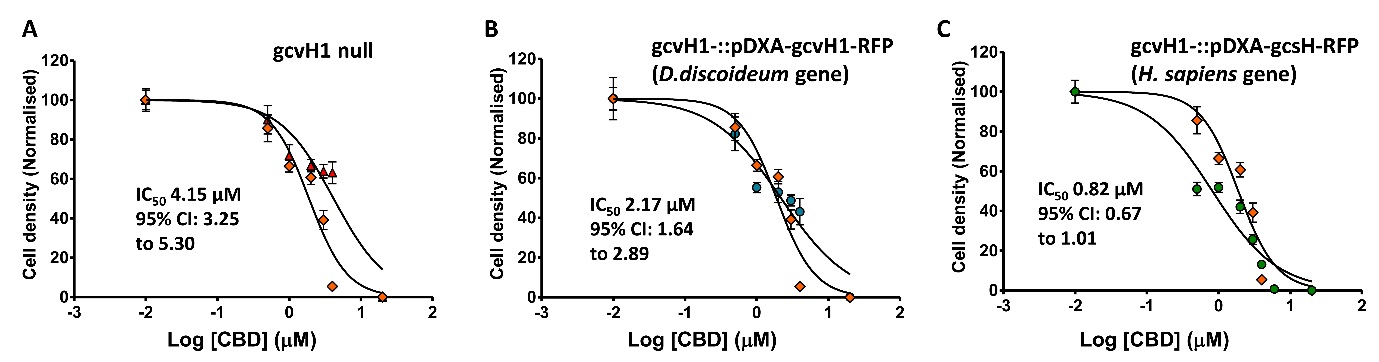
Figure S7. Dose-response curves for *gcvH1^-^* and rescue cell lines.** Comparison of CBD growth sensitivity of *D. discoideum* wild-type (orange diamond), *gcvH1*^-^ (red triangle), *gcvH1*^-^::gcvH1-RFP (blue circle), and *gcvH1*^-^::gcsH-RFP (green circle) cell lines. (A) Dose-response curves of normalised cell density against the Log (concentration) for CBD was used to calculate the IC_50_ values with 95% confidence intervals for both the *gcvH1-* and the rescue cell lines (n=6-9, experiments were repeated).
